# Ohio State Veterinary Medical Association

**Published:** 1884-10

**Authors:** J. S. Butler

**Affiliations:** Cor. Secretary


### REPORTS.

### OHIO STATE VETERINARY MEDICAL ASSOCIATION.

The semi-annual meeting of the Ohio State Veterinary Medical Association took place in the Tyndal room, City Hall, Columbus, on the 3d and 4th of September.

The meeting was called to order at 7:30 P. M. on the 3d by the president, Dr. W. C. Fair, Cleveland, who made a few remarks, and then called on the secretary, Dr. J. M. Waddel, Columbus, to call the roll, the following gentlemen answering to their names: W. C. Fair, V.S., Cleveland; J. V. Newton, V.S., Toledo; T. B. Cotton, V.S., Mount Vernon; W. A. Labron, V.S., Xenia; J. M. Waddel, V.S., Columbus; J. S. Butler, V.S., Piqua; T. B. Hillock, V.S., Columbus; L. B. Chase, V.S., Berlin; W. R. Howe, V.S., Dayton; R. W. Whitehead, V.S., Youngstown; J. C. Meyer, M.D., V.S., Cincinnati; C. C. Crane, V.S., Akron; J. B. Crane, V.S., Sharon Centre; W. Huntsberger, V.S., East Union; W. E. Wright, V.S., Delaware; A. H. Tanner, V.S., Ashtabula; J. E. Taylor, V.S., Toledo; J. B. Hillock, V.S., Lancaster.

Several new members were proposed, among whom were G. W. Butler, V.S., Circleville; L. A. Severcool, V.S., Norwalk; A. J. Smith, Pleasant Hill. The two former being graduates were admitted, and the latter gentleman referred to the Board of Censors, who, after passing a very creditable examination, was admitted.

The visiting gentlemen were, S. D. Mc Clure, V.S., Sandusky; James A. Lee and T. E. Jones, Mount Vernon; E. A. Ebersole, Fostoria; and D. E. Salmon, D.V.M., of the Bureau of Animal Industry, Washington, D. C.

The minutes of the previous meeting were then read and approved, and Dr. Salmon called upon to address the meeting, which he did upon the subject of Pleuro-Pneumonia Contagiosa and the distinguishing symptoms between it and Tuberculosis. Upon closing his address he was tendered the thanks of the meeting.

The committee appointed at a previous meeting was called upon to report on a code of ethics and the revision of the by-laws. The chairman, Dr. Cotton, stated they were not ready to report, and asked for an extension of time, which was granted.

Moved and seconded, that Dr. Meyer act on the above committee in the absence of Dr. Blanchard. Carried.

Moved and seconded, that a committee be appointed to draft resolutions of respect to the late Dr. Bowler, of Cincinnati, and a copy be sent to the widow and family of the deceased, aud also recorded upon the minutes of the Association. Carried. Drs. Newton, Butler, and Whitehead were chosen.

The meeting then adjourned until 8 A. M. the following day.

*Morning Session.*

The meeting was again called to order by Dr. Fair, and Dr. Whitehead, of Youngstown, called upon to read his Essay on Tuberculosis. It was a very able and lengthy paper, and upon closing Dr. Whitehead was tendered the thanks of the meeting.

The appointment of delegates to the National Convention was next in order, and the following gentlemen were chosen—Drs. Newton, Butler, Hillock, Severcool, and Cotton.

Moved and seconded, that an order be drawn on the treasurer for hall rent, gas, etc. Carried.

Moved and seconded, that Mr. J. Rose, Columbus, be requested by the secretary to appear at the next meeting and answer to charges preferred against him, and show cause why he should not be expelled from the Association. Carried.

Moved and seconded, that a committee of three be appointed to wait upon the State Board of Agriculture and use such steps as may be advisable in the suppression of contagious diseases, and advise the Legislature in regard to the necessity of a State Veterinarian being appointed. Carried. The following gentlemen were chosen as the committee—Drs. Cotton, Wright, and Hillock.

Moved and seconded, that the expenses of the committee be borne by the Association. Carried.

Moved and seconded, that our next meeting be held at Toledo, on the 27th of December, 1884, and that Dr. Newton be delegated to secure a place for the meeting. Carried.

Dr. Butler volunteered to read a paper on Pleuro-Pneumonia Contagiosa, and Dr. Wright one on Epizootic Cellulitis.

The meeting then adjourned.

J. S. Butler, V.S., Cor. Secretary.

